# Spatio-temporal evolution and driving factors of new urbanization in central China based on multi-source data

**DOI:** 10.1371/journal.pone.0298099

**Published:** 2024-03-11

**Authors:** Yu An, Lingtong Peng, Liang Geng

**Affiliations:** School of Science, Hubei University of Technology, Wuhan, China; East China Normal University, CHINA

## Abstract

Urbanization is an inevitable outcome of the development of human society to a certain stage, and it is also an irreversible pattern of the concentration degree of human society. Based on multi-source data such as remote sensing images, ecological environment and socio-economic data, the evaluation index system of new urbanization is constructed from multi-dimensions of population, economy, society, space and ecology. To explore the spatio temporal evolution and driving factors of urbanization in 80 prefecture-level cities in central China from 2013 to 2021 by using entropy method, spatial autocorrelation model and geographic detector. The results show that: (1) The level of new urbanization continues to grow, with the average value rising from 0.1562 in 2013 to 0.2557 in 2021, and the regional differences are obvious, forming a circle structure with Wuhan, Zhengzhou and other provincial capitals as the center and weakening radiation to surrounding cities. (2) The agglomeration of ecological urbanization is significant, and the agglomeration trend is gradually enhanced. The high-high agglomeration areas tend to Xinzhou City, most prefecture-level cities in Hubei Province and some prefecture-level cities in Southern Hunan Province, while the low-low agglomeration areas tend to Changzhi City, most prefecture level cities in Henan Province and some prefecture-level cities in Northern Anhui Province. (3) The night light index, total retail sales of consumer goods, investment in fixed assets, proportion of built-up areas and urban economic density are the main driving factors affecting the level of new urbanization. (4) The interaction of driving factors shows double factor enhancement and nonlinear enhancement effects.

## 1. Introduction

Urbanization is an important engine of modernization and economic growth, and is regarded as an inevitable trend of human social development [1]. China’s urbanization has been named as one of the two key factors affecting human development in the 21st century by Stiglitz, winner of Nobel Prize in economics [2]. Since the beginning of the 21st century, the urbanization rate of China’s permanent residents has increased from 36.22% in 2000 to 64.72% in 2021, which is far lower than the average level of developed and developing countries and has a large space for development [3]. In 2014, the state officially issued *the National New Urbanization Plan (2014–2020)*, marking the major transformation of China’s urbanization development [4]. The 19th National Congress of the Communist Party of China proposed a new urbanization strategy that focuses on promoting people’s urbanization and focuses on improving quality [5]. The 20th National Congress of the Communist Party of China continued to propose to vigorously promote the construction of new urbanization, which has become an important engine to promote China’s economic development and social progress [6]. As an important geographical region in China, central China plays a key role in the coordinated development of regional economy and plays an important role in connecting the east with the west [7]. In 2016, the National Development and Reform Commission issued *the 13th Five-Year Plan for Promoting the Rise of the Central China*, which clearly positioned the central China as a key area in the national new urbanization construction. Under the above background, it is of great significance to study the spatio-temporal evolution of new urbanization in central China and its driving factors for optimizing China’s urbanization development pattern and promoting regional coordinated and sustainable development.

At present, the definition of urbanization is not completely unified, but there is some consensus on its connotation. Scholars generally believe that urbanization is mainly characterized by population growth, economic development and spatial expansion [8–11]. It is generally believed that urbanization is a regional spatial process, which shows the spatial expansion of urban scale and form, as well as the dynamic evolution of urban functions and functions. In essence, urbanization is a coupling and coordination process between urban activities and supporting environment, and a spatial expression of man-land relationship regional system. In addition to the internal population and economic spatial agglomeration, urbanization also includes material, energy and information exchange with the background environment. Urbanization, as an evolutionary process of economic and social development, is closely related to economic level, ecological environment and social transformation [12].

Studies on urbanization mainly involve urbanization level measurement, development process, influencing factors and urban problems [13–16]. Urbanization level measurement is mainly based on single population data or the construction of evaluation index system to measure urbanization development level [17,18]. The measurement criteria of development process are composed of urbanization rate, population density and urban density [19,20]. The characteristics and dynamic evolution of urbanization pattern can be revealed through development process [21], and regional differences of urbanization levels at different scales can be analyzed [22]. The analysis of influencing factors is mostly carried out from the aspects of economic development [23,24], population migration [25], industrial structure [26], administrative policy and development planning [27]. In addition, interdisciplinary integration of urbanization studies with sociology, ecology and other disciplines focuses on the transformation from economic development to sustainable development issues such as resources, environment and rural construction [28,29]. This paper explores the sustainable development of new urbanization from the perspectives of the coupling between new urbanization and economic development, ecological environment, basic public services and other factors [30,31], urban diseases caused by urban expansion, ecological resource problems and other development problems and countermeasures [32,33]. However, the above studies pay more attention to urban development in terms of population, economy, space and social services, but not enough attention to urban ecological environment, and relatively unified views have not been formed on the selected index system. It often expresses the coordination effect between urbanization and other systems, and cannot accurately express the coordinated development process within urbanization. In addition, most studies focus on the national, provincial and municipal levels, and tend to study coordinated development from a macro perspective, while studies from the middle perspective of prefecture-level cities are rare.

Therefore, based on the panel data of 80 prefecture-level cities in the central China from 2013 to 2021, this paper constructs a comprehensive evaluation index system for new urbanization, uses entropy method to calculate index weights, and uses spatial autocorrelation model and geographic detector to explore the evolution of the spatial-temporal pattern of new urbanization in the central China and its driving factors, so as to provide reference value for the balanced development of the central China. It will provide a scientific basis for the formulation of policies for coordinated development of urbanization in relevant regions. Compared with previous studies, the innovations are as follows: Firstly, based on the mesoscopic perspective, compared with the macro perspective, mesoscopic perspective is more suitable for research on the prefecture-level city level; Secondly, the use of multi-source data, on the basis of traditional social and economic data, the introduction of satellite remote sensing image data to supplement, forming multi-source data to reflect the level of urbanization more three-dimensional; Thirdly, construct a multi-dimensional index system, and build a comprehensive evaluation index system for new-type urbanization from the five dimensions of population, economy, society, space and ecology to make up for the deficiencies of a single dimension.

## 2. Materials and methods

### 2.1 Study area

**Fig 1:**
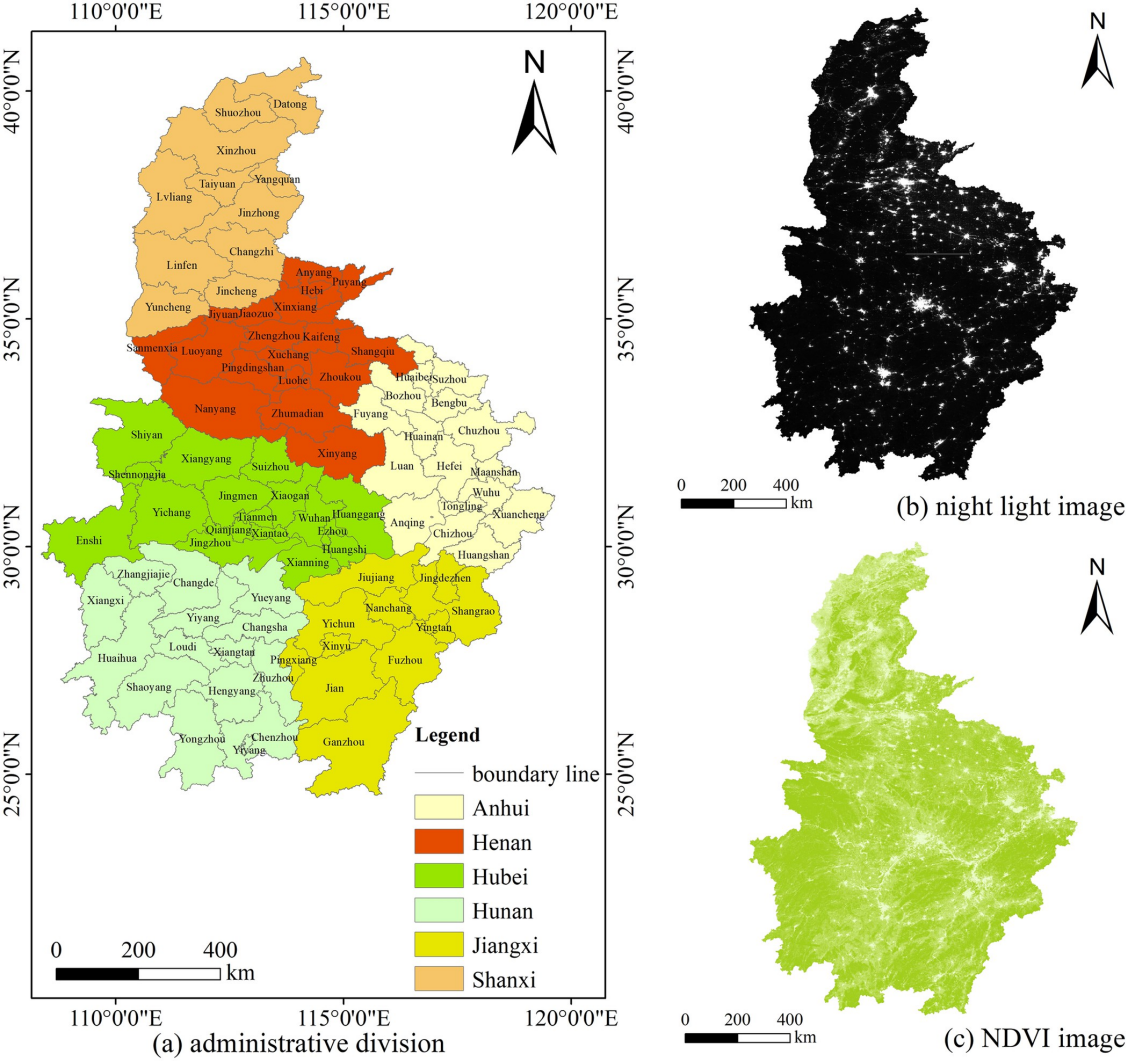
Location overview and remote sensing image of central China. Note: Based on the standard map No. GS (2016) 2556 downloaded from the National Catalogue Service For Geographic Information, the base map has not been modified.

The central China is located in the inland hinterland, including 6 neighboring provinces of Hubei, Hunan, Jiangxi, Anhui, Henan and Shanxi as well as 80 prefecture-level cities under their jurisdiction (Fig 1), playing an important role in the national regional development pattern [34]. The central China has unique geographical location conditions, superior resource endowment, and rich human resources, natural resources, culture and ecological advantages. In recent years, with the deepening of the strategy of the rise of the central China and the development of the Yangtze River Economic Belt, the central China has achieved rapid economic and social development in all aspects, gradually becoming the fourth growth pole of the country’s economic growth. By the end of 2021, the central China will have a land area of 1.03 million square kilometers, a permanent population of about 360 million and a GDP of 25 trillion yuan, accounting for 11 percent, 26 percent and 25 percent of the country’s total. In the context of accelerated development of social economy and urbanization, it is particularly timely and necessary to take 80 prefecture-level cities in central China as research samples to analyze the spatio-temporal evolution of new urbanization and its driving factors.

### 2.2 Data sources and processing

The data used in this paper mainly include administrative division vector data, ecological environment data, social and economic data, night light index (NLI) remote sensing image data, normalized difference vegetation index (NDVI) remote sensing image data. Among them, the vector data of administrative divisions came from 1:1,000,000 vector data provided by the National Catalogue Service For Geographic Information. The ecological environment and socio-economic data were obtained from the statistical yearbook, ecological environment Bulletin, national economic and social development Statistical Bulletin of provinces and prefecture-level cities in the central China from 2013 to 2021, and some data were calculated based on the yearbook data. Individual missing data were calculated by interpolation of adjacent years.

NLI and NDVI data are derived from Resource and Environment Science and Data Center of Chinese Academy of Sciences. Based on the *NPP/VIIRS* satellite noctiluminosity remote sensing image data from 2013 to 2021, the annual degree NLI data set of central China is generated with a spatial resolution of 0.004 degrees. Obviously, the intensity of light is positively correlated with socio-economic factors. This quantitative relationship can be used to study the estimation of socio-economic factors. Based on *SPOT/VEGETATION* satellite remote sensing image data, the annual NDVI data set of central China is generated by the maximum synthesis method on the basis of monthly data with a spatial resolution of 1km. The data set effectively reflected the distribution and change of vegetation cover in central China on both spatial and temporal scales. It has very important reference significance for the research of ecological environment related fields.

### 2.3 Methods

#### 2.3.1 Index system construction

Traditional studies tend to use population or economic attributes to measure the level of urbanization. From the perspective of the development of cities and towns, the development of cities and towns cannot be separated from the functions of population, space, economy and social services. While new urbanization should pay attention to the development of cities and towns themselves, it should also pay attention to the coordinated development of cities and ecology. Considering the availability, completeness and scientificity of index data, referring to relevant research results [35,36], NLI and NDVI data were introduced on the basis of previous studies, and 25 indicators were selected from the five dimensions of population, economy, society, space and ecology in combination with regional differences and the actual situation of the central China to build a new urbanization level evaluation index system (Table 1). Among them, population urbanization emphasizes the differences in population size and urbanization level among prefecture-level cities, economic urbanization highlights the differences in economic development, industrialization and per capita level among prefecture-level cities, social urbanization focuses on local fixed asset investment, social consumption and budget income levels, and spatial urbanization represents the differences in land development degree among prefecture-level cities. Ecological urbanization focuses on the difference of natural capital and ecological environment quality among prefecture-level cities. In order to eliminate the impact of population size differences among prefecture-level cities, each index adopts per capita index.

**Table 1:** New urbanization level evaluation index system.

Criterion	Index	Units	Attribute	Weight
population urbanization	urban population(*X*_1_)	people	+	0.0558
	urban population density(*X*_2_)	ten thousand people/km^2^	+	0.0577
	permanent resident urbanization rate(*X*_3_)	%	+	0.0259
	proportion of employment in secondary and tertiary industries(*X*_4_)	%	+	0.0274
economic urbanization	GDP per capita(*X*_5_)	yuan	+	0.0414
	proportion of output value of secondary industry(*X*_6_)	%	-	0.0108
	proportion of output value of tertiary industry(*X*_7_)	%	+	0.0122
	per capita disposable income of urban residents(*X*_8_)	yuan	+	0.0265
	night light index(*X*_9_)	-	+	0.0780
social urbanization	health technician(*X*_10_)	people	+	0.0650
	total retail sales of consumer goods(*X*_11_)	ten thousand yuan	+	0.0871
	investment in fixed assets(*X*_12_)	ten thousand yuan	+	0.0629
	urban registered unemployment rate(*X*_13_)	%	-	0.0146
	per capita educational expenditure(*X*_14_)	yuan	+	0.0794
spatial urbanization	proportion of built-up areas(*X*_15_)	%	+	0.1044
	urban economic density(*X*_16_)	ten thousand yuan/km^2^	+	0.0901
	per capita built-up area(*X*_17_)	m^2^	+	0.0559
	per capita urban road area(*X*_18_)	m^2^	+	0.0207
	per capita green park area(*X*_19_)	m^2^	+	0.0102
ecological urbanization	energy consumption per ten thousand yuan GDP(*X*_20_)	tce/ ten thousand yuan	-	0.0073
	urban sewage treatment rate(*X*_21_)	%	+	0.0016
	green coverage rate of built-up areas(*X*_22_)	%	+	0.0012
	per capita municipal solid waste removal volume(*X*_23_)	t	+	0.0480
	per capita industrial wastewater discharge(*X*_24_)	t	-	0.0071
	normalized difference vegetation index(*X*_25_)	-	+	0.0090

#### 2.3.2 Entropy method

The entropy method is a relatively objective multi-index weighting method. Compared with the subjective weighting method, it can better avoid the deviation caused by human factors and more accurately reflect the importance of indicators [37]. Since the meaning and dimension of each index are different, in order to make the index data comparable, the range method is used for dimensionless processing, and then the entropy method is used to calculate the weight. The calculation formula is as follows:

$$Positive\;indicators:\;Z_{ij}=\frac{X_{ij}-X_{\min}}{X_{\max}-X_{\min}}$$ (1)

$$Negative\;indicators:\;Z_{ij}=\frac{X_{\max}-X_{ij}}{X_{\max}-X_{\min}}$$ (2)

Where, *Z*_*ij*_ represents the dimensionless value of the *j* index *X*_*ij*_ of the *i* prefecture level city,and *X*_*min*_ and *X*_*max*_ are the minimum and maximum value of the *j* index respectively.

The proportion of the *j* index of the *i* prefecture-level city *P*_*ij*_:

$$P_{ij}=Z_{ij}/\sum_{i=1}^{n}Z_{ij}$$ (3)

Entropy value *e*_*j*_ of the *j* index:

$$e_{j}=-k\sum_{j=1}^{n}P_{ij}\ln P_{ij},\;k=1/\ln mn$$ (4)

Where, *e*_*j*_ is the entropy value of the *j* index, 0≤*e*_*j*_≤1; *k* is a constant; *m* is the number of prefecture-level cities and *n* is the number of indicators.

Then, the information entropy redundancy *g*_*j*_ of the *j* index:

$$g_{j}=1-e_{j}$$ (5)

$$Weight\;w_{j}\;of\;the\;j\;index:\;w_{j}=g_{j}/\sum_{j=1}^{n}g_{j}$$ (6)

#### 2.3.3 Spatial autocorrelation model

Spatial autocorrelation is the expression of spatial dependence and heterogeneity generated by geographical location or adjacency, and it is a statistical measure of the spatial distribution structure of various elements in the regional system [38,39]. Spatial autocorrelation models are divided into global spatial autocorrelation and local spatial autocorrelation. Global spatial autocorrelation is used to test whether research objects are clustered in space, and local spatial autocorrelation is used to detect spatial clustering degree.

Global spatial autocorrelation is applied to measure whether the new urbanization level in central China has significant spatial correlation, which is usually expressed by Global Moran’s *I*, and the calculation formula is as follows:

$$I=\frac{\sum_{i=1}^{n}\sum_{j=1}^{n}W_{ij}(x_{i}-{\bar{x}}_{i})(x_{j}-{\bar{x}}_{j})}{S^{2}\sum_{i=1}^{n}\sum_{j=1}^{n}W_{ij}}$$ (7)

Where, *n* is the number of prefecture-level cities; *W*_*ij*_ is the spatial weight matrix, which is 1 if adjacent and 0 if not adjacent. The *x*_*i*_ and *x*_*j*_ are the attribute values of new urbanization in central China on corresponding spatial units respectively, ${\bar{x}}_{i}$ and ${\bar{x}}_{j}$ are the attribute average value, and *S*^*2*^ is the variance of new urbanization. The value range of Global Moran’s *I* is [1,1]. If the value is positive, it indicates that the new urbanization in central China presents spatial clustering. If the value is negative, it indicates a discrete distribution. If the value is 0, it is a random distribution.

Local spatial autocorrelation is adopted to identify the spatial dependence and heterogeneity of new urbanization in central China [40], which is usually represented by Local Moran *I*. The calculation formula is as follows:

$$I=\frac{\left(x_{i}-\bar{x}\right)}{S^{2}}\sum_{i\neq j}W_{ij}(x_{i}-\bar{x})$$ (8)

If the value *I* is significantly positive, it indicates that the new urbanization level in central China presents a high-value agglomeration area. If it is significantly negative, the low value cluster area will appear. In addition, the Local Indicators of Spatial Association (LISA) cluster map is a method used for spatial data analysis, whose purpose is to identify spatial clustering patterns. According to the cluster maps of Local Moran I and LISA, the new urbanization in central China can be divided into four spatial correlation types: high-high (H-H) cluster, high-low (H-L) cluster, low-high (L-H) cluster and low-low (L-L) cluster [41].

#### 2.3.4 Geographic detector model

Geographical detector is a statistical method to detect spatial heterogeneity and reveal the driving force behind it. It is composed of risk detector, factor detector, ecological detector and interactive detector. Compared with traditional statistical methods, geographical detector can overcome the influence of multicollinearity of independent variables and solve the spatial dependence and heterogeneity caused by scale change [42,43]. The calculation formula is as follows:

$$q=1-\frac{1}{N\sigma^{2}}\sum_{i=1}^{L}N_{i}\sigma_{i}^{2}$$ (9)

Where, *q* represents the explanation degree of driving factors to the level of new urbanization, *q*∈[0,1], the greater the value, the greater the impact of the factor on urbanization, and vice versa. *L* represents the number of layers of the driving factor *X*; *N* and σ^2^ respectively represent the number of units and variance of the research object, *N*_*i*_ and *σ*_*i*_^*2*^ respectively represent the number of spatial units and variance in layer *i*.

According to the action type, interactive detection can be divided into five categories:nonlinear weakening, single factor nonlinear weakening, double factor enhancement, independent and nonlinear enhancement [44]. The interaction types are shown in Table 2.

**Table 2:** Types of two-factor interaction.

Criterion	Interaction type
*q*(*X*_*1*_∩*X*_*2*_)<min(*q*(*X*_*1*_),*q*(*X*_*2*_))	nonlinear weakening
min(*q*(*X*_*1*_),*q*(*X*_*2*_))< *q*(*X*_*1*_∩*X*_*2*_)<max(*q*(*X*_*1*_),*q*(*X*_*2*_))	single factor nonlinear weakening
*q*(*X*_*1*_∩*X*_*2*_)> max(*q*(*X*_*1*_),*q*(*X*_*2*_))	double factor enhancement
*q*(*X*_*1*_∩*X*_*2*_) = *q*(*X*_*1*_)+*q*(*X*_*2*_)	independent
*q*(*X*_*1*_∩*X*_*2*_)> *q*(*X*_*1*_)+*q*(*X*_*2*_)	nonlinear enhancement

## 3. Results

### 3.1 Analysis of spatial-temporal change characteristics of new urbanization

#### 3.1.1 Analysis of temporal evolution characteristics

**Fig 2:**
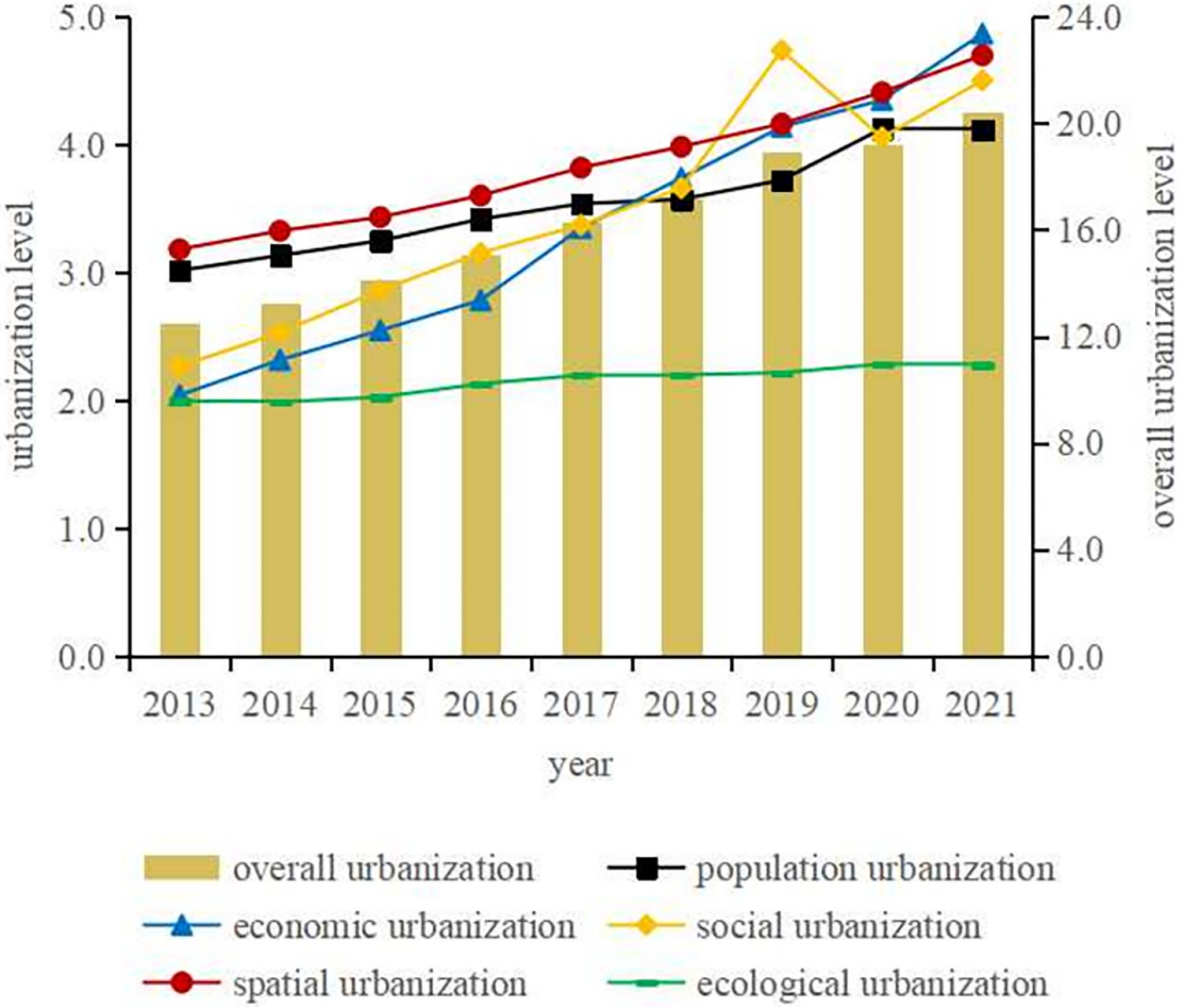
Temporal evolution of urbanization level of each dimension in central China.

According to the evaluation index system of new urbanization level in central China (Table 1),the weight is multiplied by the index standardization results to obtain the comprehensive index of urbanization level in central China from 2013 to 2021, as shown in Fig 2.

As can be seen from Fig 2, the overall urbanization level in central China continues to grow. The lowest value of the comprehensive index is 12.4969 and the highest value is 20.4565. Compared with 2013, the urbanization level in 2021 increases by 63.7%. From the perspective of all dimensions, except the social urbanization level fluctuates greatly, showing an "up-down-up" trend, the urbanization level of other dimensions shows a certain degree of stable growth. Among them, the economic urbanization level increased the most, from 2.0403 in 2013 to 4.8659 in 2021, the level of ecological urbanization is relatively stable.

#### 3.1.2 Analysis of spatial distribution characteristics

**Fig 3:**
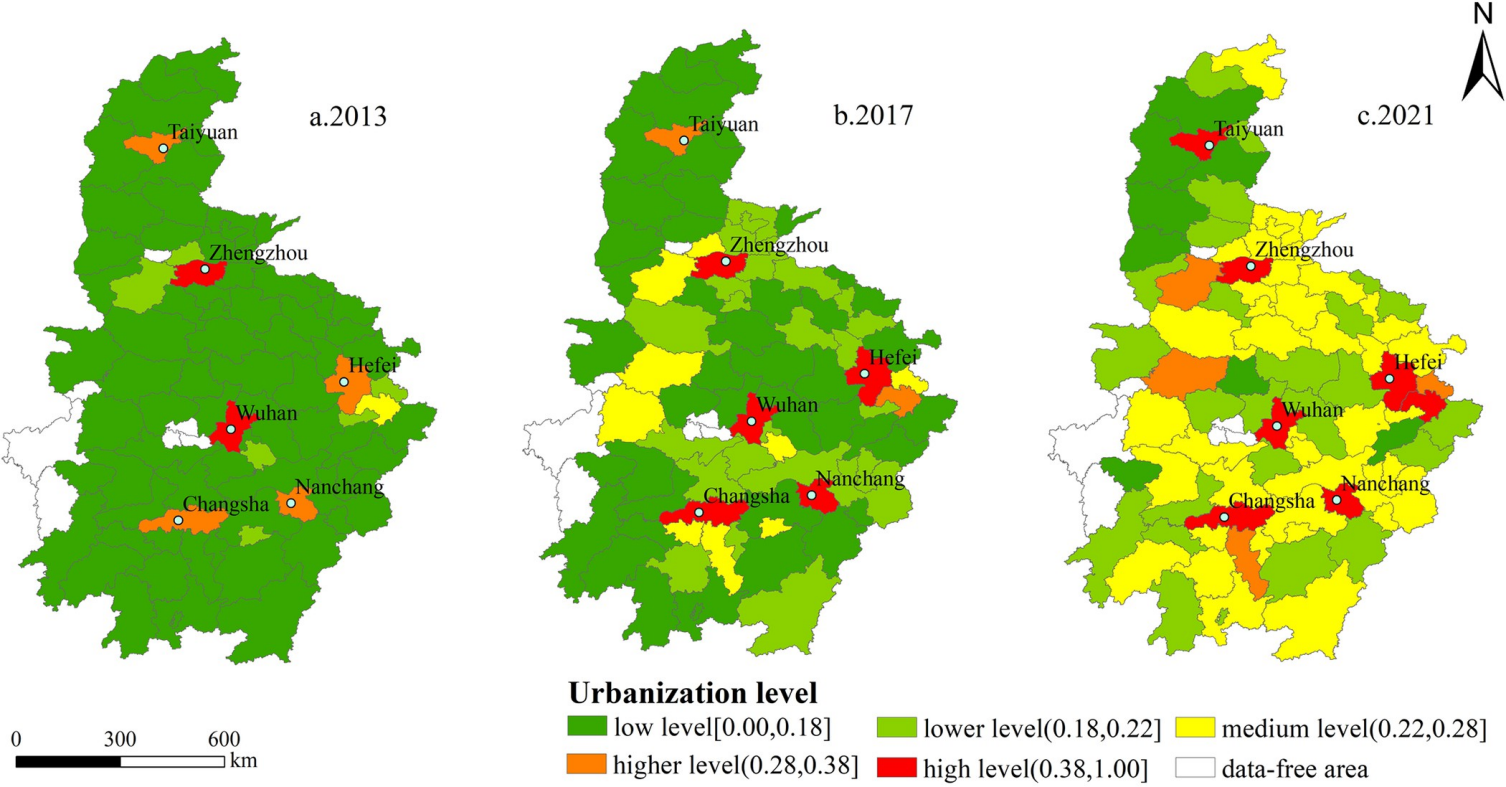
Spatial distribution of new urbanization in central China. Note: Based on the standard map No. GS (2016) 2556 downloaded from the National Catalogue Service For Geographic Information, the base map has not been modified.

According to the results of the comprehensive index of new urbanization level in central China, the natural break point method of ArcGIS10.7 was applied to divide it into five grades: high, higher, medium, lower and low (Fig 3) [45]. In 2013, the level of new urbanization was mainly low, with an average value of 0.1562. The number of prefecture-level cities at high level only accounted for 2.5%, with only Wuhan and Zhengzhou. The number of prefecture level cities at a higher level accounts for 5%, mainly distributed in provincial capitals such as Hefei, Taiyuan, Changsha and Nanchang (Fig 3A). In 2017, the overall level of new urbanization was improved to a certain extent, mainly low level and lower level, with an average value of 0.2034. The number of medium level and high level prefecture-level cities increased (Fig 3B). In 2021, the overall level of new urbanization will be further improved, with the average value of 0.2557 and the number of high level prefecture-level cities increasing to 7. However, Chizhou, Yuncheng, Jinzhong, Xinzhou, Linfen, Lvliang, Zhangjiajie and Suizhou have always been at a low level (Fig 3C). In general, the level of new urbanization in central China showed obvious regional differences during the study period, showing a circle structure with the capital cities such as Wuhan and Zhengzhou as the center and weakening radiation to surrounding cities.

### 3.2 Analysis of spatial-temporal pattern evolution characteristics of new urbanization

#### 3.2.1 Global spatial autocorrelation analysis

In order to deeply explore the evolution characteristics of the spatio-temporal pattern of new urbanization in central China, the ArcGIS10.7 software is used to analyze the spatial correlation and difference of regional urbanization through the Global spatial autocorrelation method, and the Global Moran’s *I* and test results of new urbanization in central China are calculated (Table 3).

**Table 3:** Results of Global Moran’s *I* of urbanization in central China.

Urbanization system	2013		2017		2021	
	Moran’s *I* index	*P* value	Moran’s *I* index	*P* value	Moran’s *I* index	*P* value
population urbanization	0.0480	0.4270	0.0454	0.4461	0.1388	0.0498
economic urbanization	0.0875	0.1861	0.0578	0.3515	0.0604	0.3399
social urbanization	-0.0416	0.6911	-0.0104	0.9755	-0.0321	0.7962
spatial urbanization	0.0698	0.2887	0.0378	0.5073	0.0813	0.2115
ecological urbanization	0.2119	0.0038	0.2686	0.0004	0.3539	0.0000
overall urbanization	-0.0068	0.9377	-0.0210	0.9099	-0.0295	0.8203

As can be seen from Table 3, the *P* values of population, economy, society, space and overall urbanization are almost all greater than 0.05, failing the 95% significance test, indicating that the spatial structure of new urbanization in central China tends to be random distribution in these dimensions, and the regional division of labor and cooperation are not obvious, and the spatial agglomeration is not significant. The Moreland index of ecological urbanization is positive and shows an upward trend through 95% significance test, indicating that there is a significant spatial agglomeration of ecological urbanization, that is, prefecture level cities with higher ecological urbanization tend to cluster relatively, while prefecture level cities with lower ecological urbanization tend to cluster relatively, and the agglomeration trend is gradually increasing. This is due to the persistence of ecological civilization construction in central China in the past decade. Therefore, it is necessary to further analyze the spatial dependence and heterogeneity characteristics of ecological urbanization.

#### 3.2.2 Local spatial autocorrelation analysis

**Fig 4:**
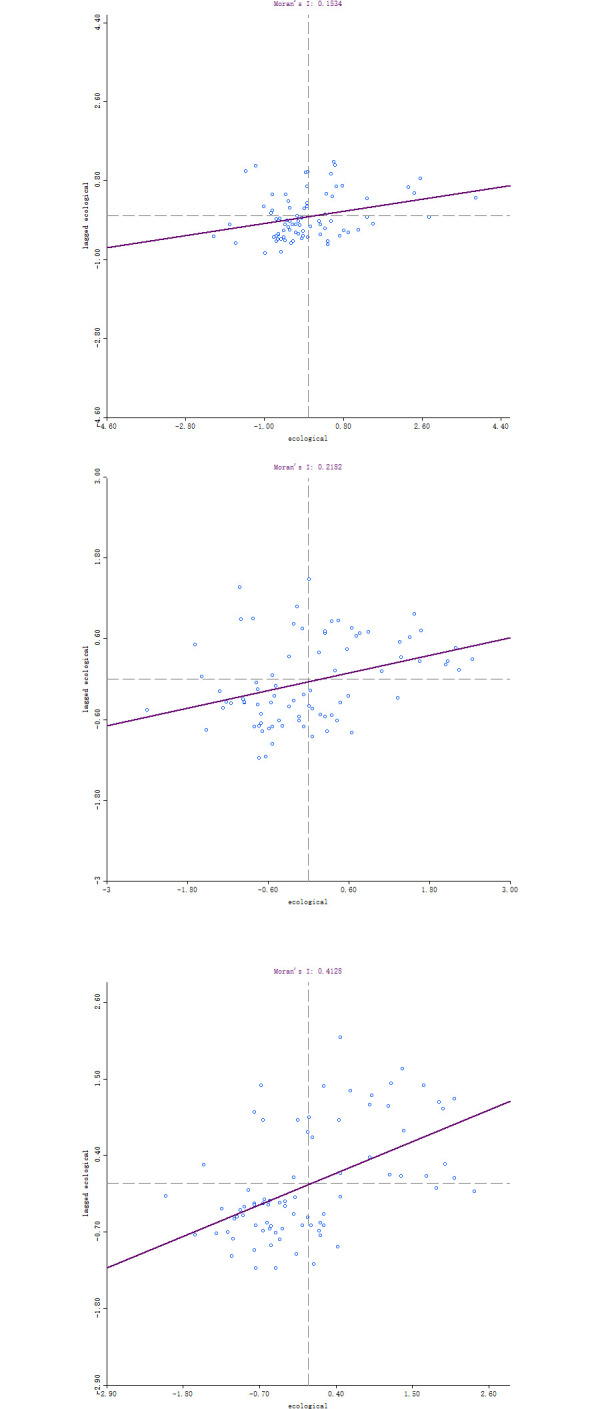
Moran’s *I* scatter chart of ecological urbanization in central China.

GeoDa095 software was used to calculate the Local Moran’s *I* value of ecological urbanization in central China from 2013 to 2021, and Moran’s *I* scatter diagram was drawn (Fig 4). From the perspective of local spatial autocorrelation, Moran’s *I* values of ecological urbanization were all positive during the study period, and scattered data were concentrated in the first and third quadrants, indicating that the new urbanization in central China formed a relatively stable spatial pattern in terms of ecology.

            a.2013                b.2017                c.2021.

**Fig 5:**
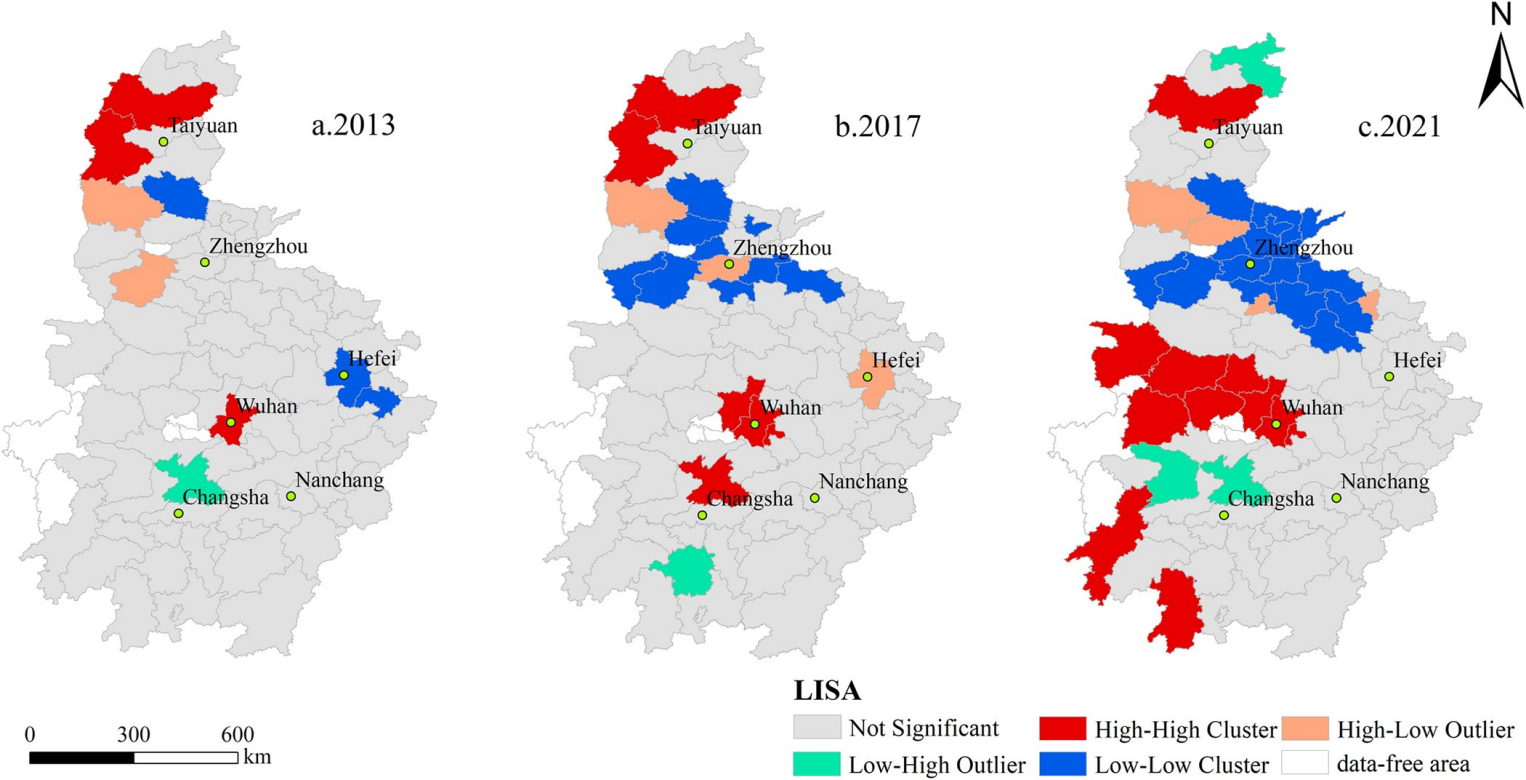
LISA cluster of ecological urbanization in central China. Note: Based on the standard map No. GS (2016) 2556 downloaded from the National Catalogue Service For Geographic Information, the base map has not been modified.

In order to more accurately measure the temporal and spatial evolution characteristics of ecological urbanization, the LISA agglomeration of ecological urbanization was drawn (Fig 5). From 2013 to 2021, the spatial pattern of ecological urbanization in central China showed obvious agglomeration characteristics of high value and low value. The high-high agglomeration areas are expanding from Wuhan, Xinzhou and Lvliang to Xinzhou, most prefecture-level cities in Hubei and some prefecture level cities in southern Hunan. The spatial difference of ecological urbanization level is small, and the ecological urbanization level of itself and neighboring prefecture-level cities is higher, showing a significant positive correlation. The low-high agglomeration areas are scattered and have large spatial differences. Due to their low ecological environment quality, the development of ecological urbanization is limited, resulting in lower ecological urbanization than neighboring prefecture-level cities. The high-low concentration areas are scattered, and the ecological urbanization level is higher than that of the neighboring prefecture-level cities due to the rational development of ecological resources, and there is a significant negative correlation with the surrounding prefecture-level cities. Low-low agglomeration areas continue to expand, and eventually tend to Changzhi, most prefecture-level cities in Henan and some prefecture-level cities in northern Anhui. Due to the combined effect of backward development and adverse natural conditions, regional ecological urbanization lacks power, and the ecological urbanization level of its own and neighboring prefecture-level cities is low.

### 3.3 Analysis of driving factors of new urbanization

By analyzing the spatio-temporal evolution characteristics of new urbanization in central China, it is found that the spatial dependence and heterogeneity of new urbanization are not significant, and it is difficult to accurately identify its driving factors by traditional statistical methods. As an effective tool to identify spatial heterogeneity features, geographic detectors can better analyze the causality generated by spatial correlation, diagnose and solve spatial heterogeneity and its causes [46]. This paper mainly uses factor detector and interaction detector to explore the influence degree of each driving factor on new urbanization in central China and the influence degree of each factor interaction.

#### 3.3.1 Factor detection analysis

The driving factors of the new urbanization system are classified and sorted by k-means clustering method [47]. With the urbanization level as the dependent variable, population, economic, social, spatial and ecological urbanization as the independent variables, combined with the geographic detector model, the driving factors of the new urbanization level in central China are detected and analyzed by Geodetector software. The results are shown in Table 4.

**Table 4:** Detection results of driving factors of urbanization criterion layer in central China.

Driving factor	2013		2017		2021	
	*q* value	*P* value	*q* value	*P* value	*q* value	*P* value
population urbanization	0.821	0.000	0.808	0.000	0.573	0.062
economic urbanization	0.769	0.000	0.892	0.000	0.855	0.000
social urbanization	0.737	0.000	0.791	0.000	0.791	0.000
spatial urbanization	0.772	0.000	0.788	0.000	0.771	0.000
ecological urbanization	0.119	0.967	0.075	0.618	0.100	0.314

As can be seen from Table 4, during the study period, population, economic, social and spatial urbanization almost all passed the 95% significance test, while only ecological urbanization failed the test. From the perspective of the development of cities and towns themselves, the development of urbanization cannot be separated from the functions of population, space, economy and social services, although the new urbanization also focuses on the coordinated development of cities and ecology. However, China’s ecological civilization construction started late, and the construction of ecological urbanization in central China has only been more than ten years, so the driving effect of ecological urbanization on the new urbanization is weak. The *q* value of urbanization in each dimension has gradually changed from population urbanization > spatial urbanization > economic urbanization > social urbanization in 2013 to economic urbanization > social urbanization > spatial urbanization > population urbanization in 2021. This indicates that the focus of determining the level of new urbanization has shifted from the basic level of population and spatial urbanization to the high-level and advanced level of economic and social urbanization.

Further, the specific driving factors and their interaction relationship were explored for 25 secondary indexes, and the results were shown in Table 5. In 2013, the *q* value of *X*_15_ ranked first among all index factors, indicating that the proportion of built-up areas was the main driving force for promoting urbanization development. The *q* values of *X*_16_, *X*_9_, *X*_11_ and *X*_12_ exceed 0.7 and pass the 95% significance test, indicating that urban economic density, night light index, total retail sales of consumer goods and investment in fixed assets have important driving effects on urbanization. The value of other index factors *q* was less than 0.7, and part of them failed the significance test, showing weak explanatory ability. In 2017, the *q* value of urban economic density *X*_16_ jumped to the first place. The *q* values of *X*_1_, *X*_5_, *X*_9_, *X*_10_, *X*_11_, *X*_12_ and *X*_15_ are greater than 0.7, and pass the 95% significance test, indicating that the enhancement of economic and social urbanization has promoted the improvement of urbanization level. The explanatory ability of other index factors is weak. In 2021, the *q* value of *X*_16_ of urban economic density still ranks first. The *q* values of *X*_1_, *X*_9_, *X*_10_, *X*_11_, *X*_12_ and *X*_15_ are greater than 0.7, and pass the 95% significance test, indicating that economic and social urbanization is an important driving force to promote urbanization development. The explanatory ability of other index factors is weak. In general, during the study period, night light index *X*_9_, total retail sales of consumer goods *X*_11_, investment in fixed assets *X*_12_, proportion of built-up areas *X*_15_ and urban economic density *X*_16_ are the main driving factors affecting new urbanization in central China.

**Table 5:** Detection results of driving factors of urbanization index layer in central China.

Driving factor	2013		2017		2021	
	*q* value	*P* value	*q* value	*P* value	*q* value	*P* value
*X*_1_	0.674	0.000	0.749	0.000	0.790	0.000
*X*_2_	0.219	0.952	0.067	0.879	0.041	0.747
*X*_3_	0.589	0.000	0.577	0.000	0.635	0.000
*X*_4_	0.654	0.000	0.668	0.000	0.444	0.000
*X*_5_	0.682	0.000	0.716	0.000	0.545	0.000
*X*_6_	0.129	0.115	0.026	0.910	0.034	0.834
*X*_7_	0.048	0.910	0.194	0.270	0.390	0.000
*X*_8_	0.575	0.000	0.471	0.044	0.509	0.013
*X*_9_	0.812	0.000	0.777	0.000	0.765	0.000
*X*_10_	0.648	0.008	0.706	0.005	0.765	0.000
*X*_11_	0.749	0.000	0.843	0.000	0.821	0.000
*X*_12_	0.717	0.000	0.799	0.000	0.780	0.000
*X*_13_	0.073	0.529	0.030	0.857	0.039	0.871
*X*_14_	0.107	0.588	0.384	0.587	0.106	0.307
*X*_15_	0.814	0.000	0.822	0.000	0.804	0.000
*X*_16_	0.812	0.000	0.856	0.000	0.853	0.000
*X*_17_	0.378	0.038	0.369	0.019	0.283	0.022
*X*_18_	0.061	0.608	0.095	0.656	0.068	0.635
*X*_19_	0.023	0.944	0.044	0.964	0.038	0.923
*X*_20_	0.035	0.827	0.056	0.615	0.092	0.240
*X*_21_	0.037	0.903	0.045	0.676	0.022	0.942
*X*_22_	0.026	0.966	0.030	0.867	0.035	0.901
*X*_23_	0.160	0.508	0.185	0.210	0.187	0.733
*X*_24_	0.559	0.040	0.653	0.004	0.612	0.000
*X*_25_	0.332	0.013	0.267	0.053	0.405	0.027

#### 3.3.2 Interaction detection analysis

**Fig 6:**
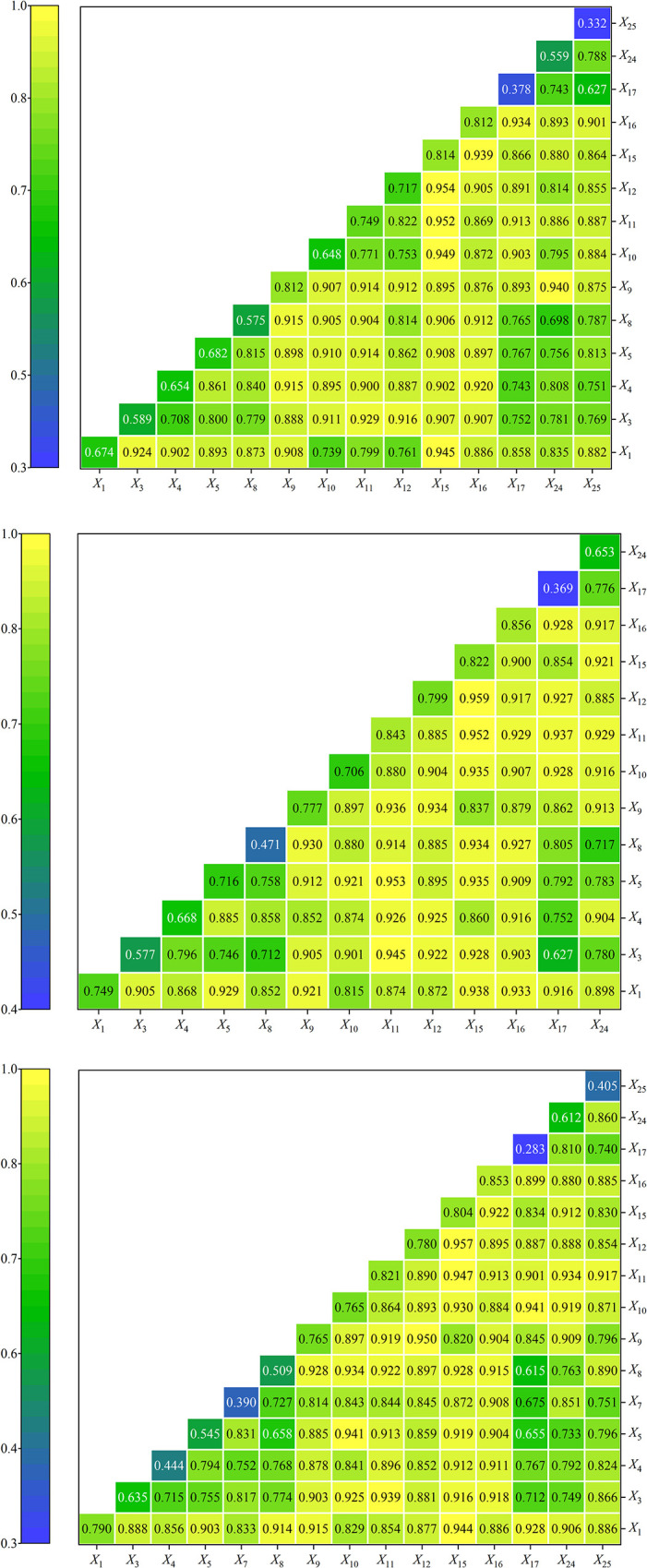
Interactive detection results of driving factors of urbanization in central China.

In order to further explore the interaction relationship among driving factors of new urbanization in central China, the index factors that failed the test were removed at the significance level of 5%, and the interaction detector was used to analyze the influence degree of the interaction of driving factors on new urbanization (Fig 6). In 2013, the interaction between *X*_12_ and *X*_15_ was the strongest, with *q* value of 0.954. *X*_11_ and *X*_15_, *X*_10_ and *X*_15_, *X*_1_ and *X*_15_, and *X*_9_ and *X*_24_ had strong interaction, and *q* values were all above 0.9, indicating that the influence of dual-factor interaction on urbanization level was enhanced. In 2017, the interaction between *X*_12_ and *X*_15_ was the strongest, with *q* value of 0.959. *X*_5_ and *X*_11_, *X*_11_ and *X*_15_, *X*_3_ and *X*_11_ are stronger, indicating that their synergistic effect is conducive to the promotion of urbanization level. In 2021, *X*_12_ and *X*_15_ have the strongest influence with *q* value of 0.957, while *X*_9_ and *X*_12_, *X*_11_ and *X*_15_, *X*_1_ and *X*_15_, *X*_10_ and *X*_17_, and *X*_5_ and *X*_10_ are stronger, indicating that their synergistic effect is an important driving force for urbanization development. To sum up, the interaction of driving factors during the study period presents double factor enhancement and nonlinear enhancement effects, and the *q* values of the interaction of any two factors on new urbanization are significantly greater than the single factor independent effect, indicating that the double factor interaction will enhance the interpretation of urbanization level.

            a.2013            b.2017            c.2021

## 4. Conclusion and discussion

Based on the mesoscopic perspective of prefecture-level cities and multi-source data such as remote sensing images, ecological environment and social economy, this paper constructs a new urbanization evaluation index system in multiple dimensions, uses entropy method to calculate index weights, and adopts spatial autocorrelation model and geographic detector to explore the spatio-temporal evolution characteristics and driving factors of urbanization level in 80 prefecture-level cities in central China from 2013 to 2021. The main conclusions are as follows:

1. There are obvious regional differences in urbanization, showing a circular structure with Wuhan, Zhengzhou and other provincial capitals as the center and a weakening radiation to surrounding cities. During the study period, the new urbanization level in central China continued to rise, with a growth rate of 63.7%. Social urbanization showed an "upward downward-upward" trend, and economic urbanization had the largest growth rate. The average level of regional urbanization increased from 0.1562 in 2013 to 0.2557 in 2021, generally at a medium and lower level, while Chizhou, Yuncheng, Jinzhong, Xinzhou, Linfen, Lvliang, Zhangjiajie and Suizhou were always at a low level.
2. The agglomeration of ecological urbanization is significant, and the agglomeration trend is gradually enhanced, the spatial pattern of high value and low value agglomeration is obvious. During the study period, the Moran’s *I* index of ecological urbanization in central China increased from 0.2119 in 2013 to 0.3539 in 2021, while the spatial structure of urbanization in other dimensions tended to be random distribution, and agglomeration was not significant. The high-low and low-high ecological urbanization agglomeration areas are scattered. High-high agglomeration areas are expanding, mainly distributed in Xinzhou, most prefecture-level cities in Hubei Province and some prefecture-level cities in southern Hunan Province. Low-low agglomeration areas tend to Changzhi, most prefecture-level cities in Henan province and some prefecture-level cities in northern Anhui Province.
3. Night light index, total retail sales of consumer goods, investment in fixed assets, proportion of built-up areas and urban economic density are the main driving factors affecting urbanization level. During the study period, the *q* values of night light index, total retail sales of consumer goods, investment in fixed assets, proportion of built-up areas and urban economic density are all greater than 0.7. The focus of determining the level of new urbanization in central China has shifted from the population and spatial levels to the economic and social levels. The enhancement of economic and social urbanization has promoted the improvement of new urbanization level.
4. The interaction of driving factors showed double factor enhancement and nonlinear enhancement effects. During the study period, the interaction of two factors will enhance the interpretation of the level of new urbanization, and the *q* value of any interaction of two factors is greater than the independent effect of single factor, and the synergistic effect will help promote the improvement of the level of new urbanization.

The research results of this paper are helpful to clarify the evolution trend and driving mechanism of urbanization in central China, and provide reference value for the balanced development of the central China. At the same time, the article still has some shortcomings.

1. In terms of research scale, the analysis is mainly based on the mesoscopic perspective of prefecture-level cities, and there is no more detailed research on the level of urbanization at the micro-scale county level, which needs to be improved in the future.
2. In terms of index selection, Multi-source data such as remote sensing images, ecological environment and social economy data are combined for selection, but the uniformity and representativity of the indicators need to be further enriched and improved.
3. In terms of research content, different urbanization levels of prefecture-level cities can be further classified and zoned in the future, so as to better provide systematic and targeted reference information for the new urbanization development of different prefecture-level cities, and thus improve the application value of the research results.

## Supporting information

S1 Data (XLS)
